# Supporting Aboriginal and Torres Strait islander cultural educators and cultural mentors in Australian general practice education

**DOI:** 10.1186/s12909-018-1340-x

**Published:** 2018-10-11

**Authors:** Jennifer Reath, Penelope Abbott, Linda Kurti, Ruth Morgan, Mary Martin, Ada Parry, Elaine Gordon, Julian Thomas, Marlene Drysdale

**Affiliations:** 1 Department of General Practice, Western Sydney University, Campbelltown, Australia; 2 Department of General Practice, Western Sydney University, Sydney, Australia; 3 URBIS, Sydney, Australia; 4 Western Sydney University, Sydney, Australia; 5 Queensland Aboriginal and Islander Health Council, South Brisbane, Australia; 6 Menzies School of Health Research, Darwin, Australia; 7 Murray City Country Coast GP Training, Melbourne, Australia

**Keywords:** Aboriginal and Torres Strait islander, Cultural, Mentoring, Education, General practice, Registrar training

## Abstract

**Background:**

Promoting cultural competence of health professionals working with Aboriginal and Torres Strait Islander communities is key to improving health outcomes. Cultural Educators and Cultural Mentors (CE/ CMs) have critical roles in Australian general practice training, yet these are not well understood.

**Methods:**

Guided by a CE/CM Network, our research team including experienced CE/CMs, used surveys and semi-structured interviews to explore these roles and investigate best practice in employment and support. Participants sampled from stakeholders involved in general practice education across Australia included CE/CMs, Medical Educators, General Practice Supervisors and Registrars, and representatives of Regional Training Organisations, Indigenous Health Training Posts and other key organisations. We undertook thematic analysis using a framework approach, refined further in team discussions that privileged views of CE/ CM members.

**Results:**

Participants comprised 95 interviewees and 55 survey respondents. We organised our findings under three overarching themes: understandings about cultural education and mentoring; employment and support of CE/CMs; and delivery and evaluation of cultural education and mentoring. Our findings supported a central role for Aboriginal and Torres Strait Islander CE/CMs in face-to-face Registrar education about culture and history and related impacts on health and healthcare. Cultural education was reported to provide base-line learning as preparation for clinical practice whilst cultural mentoring was seen as longitudinal, relationship-based learning. Mentoring was particularly valued by Registrars working in Aboriginal and Torres Strait Islander communities.

Challenges described with employment and support included difficulties in finding people with skills and authority to undertake this demanding work. Remuneration was problematic, particularly for CMs whose work-time is difficult to quantify, and who are often employed in other roles and sometimes not paid. Other improved support recommended included appropriate employment terms and conditions, flexibility in role definitions, and professional development. Recommendations concerning implementation and evaluation included valuing of cultural education, greater provision of mentoring, partnerships with Medical Educators, and engagement of CE/CMs in rigorous evaluation and assessment processes.

**Conclusions:**

Our research highlights the importance of the unique CE/CM roles and describes challenges in sustaining them. Professional and organisational support is needed to ensure delivery of respectful and effective cultural education within general practitioner training.

## Background

Similar to other Indigenous peoples, Aboriginal and Torres Strait Islander people, who constitute 3.1% of the Australian population, suffer worse outcomes in almost all health indicators compared to non-Indigenous Australians [1, 2]. Social determinants of health underlay much of this inequity and require cross sectoral action [3], however reduction and management of the disease burden require access to culturally appropriate health services [4]. Building the cultural competence of health professionals so that they can provide appropriate and effective healthcare with Aboriginal and Torres Strait Islander peoples is an important strategy and one which has been strongly endorsed by Aboriginal and Torres Strait Islander people [5, 6] and is supported by international research [7].

In alignment with calls for training in cultural diversity to be introduced in undergraduate medical teaching internationally [8], Australian medical schools and most professional colleges have compulsory learning requirements in Aboriginal and Torres Strait Islander health [9, 10]. In Australia general practitioners (GPs) are the first port of call for many health problems and act as gate-keepers to other medical care. Training in Aboriginal and Torres Strait Islander health has been a focus of general practice vocational training for the past 20 years [11]. Key achievements include general practice training curricula and assessment and practice standards that address Aboriginal and Torres Strait Islander health [10, 12].

In accordance with these standards, training in this area has been delivered largely in partnership with Aboriginal and Torres Strait Islander people and communities, with individuals selected for educational roles based on their expertise in cultural issues and their leadership roles in their communities. They are described as Cultural Educators (CEs) or Cultural Mentors (CMs) and they often work closely with Medical Educators (GPs working as Registrar trainers) either in regional organisations providing postgraduate GP training (RTOs) or in Indigenous Health Training Posts where general practice registrars (Registrars) undertake clinical placements. Indigenous training posts are health services serving Aboriginal and Torres Strait Islander communities, mostly operating as Aboriginal Community Controlled Health Services [13]. Registrar teaching in these training posts is supported by GP Supervisors who also work within the health services.

Currently there are approximately 70 CE/CMs employed in general practice training in Australia. Cultural Mentors are usually community appointed (and often already in health roles) and are not required to have particular qualifications, whilst CEs often will have some education enabling them to provide more formal education sessions. Those recruited to these roles are Aboriginal or Torres Strait Islander people and range from young academics to older community Elders and Traditional Owners. Their remuneration varies from approximately $(AUD) 60/h for CMs to $80/ h for CEs (personal communication, Marlene Drysdale 2018).

The contribution of CEs and CMs to general practice training is recognised and their expertise increasingly sought in cultural education as well as in clinical teaching and assessment [11]. However there are gaps in our understandings about their roles and responsibilities, as well as what is best practice in engaging and supporting CEs and CMs [11, 14, 15].

In this paper we summarise the findings of research commissioned by Australian General Practice Training, Ltd., the national organization that oversaw GP training at the time of this research. Whilst in a comprehensive research report [16] we addressed research questions developed by a national network of CEs and CMs who oversaw the research (Table 1), in this paper we focus on those findings that build an understanding about the roles of CEs and CMs and the support required to sustain these roles. This research not only informs general practice training in Australia, but proposes ways of engaging with Indigenous cultural experts in teaching and in educational research which is likely to be relevant in other settings.

**Table 1:** Research Questions and Methodology

Research Questions	Methodology/ Data collection methods	Stakeholders consulted/ interviewed
Understanding Cultural Education and Mentoring		
1. What is currently understood to be cultural education?	Literature review. Survey, interviews and focus groups	All stakeholders comprising: Training organisations, Indigenous Health Training Posts including Aboriginal Community Controlled Health Services, CE and CM network, the National Aboriginal Community Controlled Health Organisation and its State and Territory Affiliates, Royal Australian College of General Practitioners, Australian College of Rural and Remote Medicine, Australian Indigenous Doctors Association, Indigenous General Practice Registrar Network.
2. What is currently understood to be cultural mentoring?		
3. What practices are currently used to establish positive relationships with Aboriginal and Torres Strait Islander peoples and communities? What is needed to engage and establish partnerships?	Survey and interviews	
Current Capacity and Roles		
4. When are CEs and CMs employed or engaged in the development, delivery and evaluation of General Practice training?	Survey and interviews	Training organisations, Indigenous Health Training Posts including Aboriginal Community Controlled Health Services, CE and CM network, the National Aboriginal Community Controlled Health Organisation and its State and Territory Affiliates
6. How are CEs and CMs remunerated?		
7. Do GP training organisations have formal policies in place in relation to supporting CEs and CMs?		
8. Are CEs and CMs provided with opportunities to participate in professional and cultural support and development?		
9. Do general practice training organisations run cultural education activities for staff, including Medical Educators and GP Supervisors?		
10. Are Registrars required to prepare for working in Aboriginal and Torres Strait Islander communities?	Review curricula for Registrar training, survey and interviews.	Training organisations, Indigenous Health Training Posts including Aboriginal Community Controlled Health Services, CE and CM network, the National Aboriginal Community Controlled Health Organisation and its State and Territory Affiliates, Royal Australian College of General Practitioners, Australian College of Rural and Remote Medicine
11. Are Registrars required to undertake formal cultural awareness training during general practice training?		
12. How is Aboriginal and Torres Strait Islander health incorporated into current General Practice education and training practices?	Survey and interviews.	Training organisations, Indigenous Health Training Posts including Aboriginal Community Controlled Health Services, CE and CM network, the National Aboriginal Community Controlled Health Organisation and its State and Territory Affiliates
13. Are there formal processes for feedback on cultural education and/or cultural mentoring activities?	Survey and interviews	
14. Are there feedback mechanisms for Registrars who undertake an Indigenous health training placement, and vice-versa?	Survey and interviews	
Needs Assessment		
15. What is needed to build sustainable cultural education and cultural mentoring capacity to meet Registrar training needs?	Literature review Survey and interviews.	All stakeholders (as above).
16. What is needed to build partnerships with Aboriginal and Torres Strait Islander peoples and communities to sustain Registrar training?	Focus groups and interviews	

## Methods

Our research was strongly supported by Aboriginal organisations with letters of support received from the National Aboriginal Community Controlled Health Organisation and its state and territory based affiliates. It was also approved by five Aboriginal Human Research Ethics Committees as well as the university ethics committee. The research team comprised three experienced CE/CMs (MM, AP, EG), two academic GPs (JR, PA) who also worked as clinicians and educators in Aboriginal Community Controlled Health Services, two consultants (LK, JT) and three university-based academics (RM, BA, RB), one of whom was Aboriginal. The team was supported by a national reference group of senior Aboriginal CE/CMs appointed by the funding organisation. This reference group approved the research plan including selection and recruitment of participant groups, also data collection tools and received regular updates on the progress of the research. Though they did not contribute to analysis and interpretation of the data, this group reviewed and approved the final research report.

We used a mixed methods approach collecting qualitative and quantitative data from surveys and semi-structured interviews which were adapted for the different stakeholder groups (Table 2). The use of survey and interview data and the range of participant groups provided opportunities for triangulation of data sources [17]. The development of these tools was informed by review of peer reviewed literature published in the 10 years prior to the research, and of grey literature including reports from websites of key organisations and clearing houses. Informed consent was obtained from all participants including for de-identified excerpts to be reported/ published.

**Table 2:** Survey and Interview Participants

Stakeholder Group	Survey respondents	Interviewees
Training organisation delegate	7	13
Cultural Educators and Cultural Mentors	10	27
Medical Educators		10
General Practice Registrars	36	9
Indigenous health training post delegates	2	4
GP Supervisors and Medical Directors of Indigenous Health Training Posts		9
Workshop attendees including Cultural Educators and Cultural Mentors and Medical Educators	59	

Stakeholder Organisations - 18 telephone interviews with representatives of: National Aboriginal Community Controlled Organisation and all of its state and territory affiliates, Australian Indigenous Doctors Association, GP Registrar Association, Indigenous GP Registrar Network, Royal Australian College of General Practitioners, Australian College of Rural and Remote Medicine, Aboriginal Health College, National Aboriginal and Torres Strait Islander Health Worker Association, Kaiela Institute

### Surveys

The surveys were piloted over a two month period with stakeholders related to two of the 17 RTOs operating at that time. After minor modifications these were circulated by email to all 17 RTOs with a request for a senior manager to complete the relevant survey on behalf of the organisation and to distribute other surveys to CEs, CMs, Medical Educators and to Indigenous Health Training posts. A link to an on-line survey was circulated by the national General Practice Registrar Association. Surveys were also emailed by the national CE/CM Network to their members. Survey questions were preceded by a statement explaining the purpose of the research and the use of the data and notifying respondents that their completion of the survey would be taken as consent to participate in the research (See Appendix 1). Following the pilot, surveys were in circulation for 3 months and over that time multiple telephone and email reminders were provided through the networks described, as well as through personal contact by the research team. All survey responses were de-identified, though stakeholder group and RTO affiliations were recorded.

### Interviews

Interviews were conducted over a four month period, during visits to six regions purposively selected from the 17 RTOs operating at that time, by two or three members of the research team, usually including one of the CE/ CM team members. The six regions we visited were selected for variation in geography, size of the Aboriginal and Torres Strait Islander population in their region and innovation in this area of training. The initial two site visits were used to pilot the interview guides which were subsequently aggregated into one interview guide (Appendix 2). We also interviewed representatives of seven other selected RTOs, mostly by telephone, to allow sampling from additional regions across Australia. For each RTO region, interviews were conducted with management or their delegate, Medical Educators and other staff employed by the RTO, CE/CMs working with the RTO as well as staff of related Indigenous Health Training Posts, GP Supervisors and Registrars. Identification of potential participants was facilitated by each RTO, who invited relevant staff, Supervisors and Registrars to participate and scheduled interview times or passed potential participant details to the researchers for later contact.

In addition to frequent email reminders, recruitment of interviewees was boosted through advertising at the end of surveys and at two national CE/CM meetings, as well as through snowballing, whereby contacts were asked to forward interview requests to others working in the area.

Interviews explored understandings of cultural education and mentoring, roles and challenges for CE/CMs, CE/CM training and support, and availability, content and evaluation of cultural training programs. These were generally conducted with a single participant though in some cases, where the preference of the interviewee was for a group interview, this approach was taken. Towards the end of the data collection period we conducted a large group discussion with 59 CE/CMs and Medical Educators attending a national workshop. The research team developed a discussion guide in order to explore issues where limited data had been collected up to that time and to seek participant views on themes emerging as key research findings (Appendix 3). The lead researcher (JR) presented early findings of the research in a plenary session and then participants gathered in groups including CE/CMs and Medical Educators, in which they were asked to discuss one of the two sets of questions in the discussion guide. A facilitator was appointed for each group, notes of the discussions were recorded and groups presented the results of their discussions in a following plenary session. Written informed consent was received from all interviewees including workshop attendees.

In addition, we conducted telephone interviews with representatives of key stakeholder organisations identified as having important roles and perspectives on GP training in Aboriginal and Torres Strait Islander health (Table 2).

### Data analysis

The qualitative data derived from the surveys was added to the data collected in interviews and both sets of data coded together within the thematic analysis framework described below.

All interviews but one were recorded and notes were taken during the interview. Most interviews were transcribed verbatim, with audiotape recordings of a smaller number of interviews directly analysed. Data were provided in an email response from one individual to interview questions. For the larger group discussion notes from the small groups were collected and included with notes taken by research team members (JR, PA, RM), for qualitative analysis.

We undertook data analysis of both qualitative survey data and interview data using a framework approach [18] based on categories assigned a priori according to the research objectives (Table 1). As described by Pope et al. [18] we increased rigour by engaging three researchers [JR, PA, RM] in review of and familiarisation with the data, in order to identify recurring themes. These were applied systematically to all the data which were charted to align with the themes. At two face-to-face meetings including academic researchers and CE/CM consultants, this “mapping” was reviewed and interpretations tested and contested until an agreed interpretation of the data was reached, that addressed the research objectives. This analysis took place concurrently with ongoing data collection enabling us to refine our interview guide for subsequent interviews and develop the analysis in a team approach.

The views of the CE/CM research team members were privileged at our research meetings and their endorsement of interpretations by non-Indigenous researchers was sought before these were accepted. The final analysis and report of our findings was carefully reviewed and checked not only by CE/ CM members of the research team, but also by the national Reference Group of senior CEs/ CMs in alignment with principles of decolonising research with Indigenous people [19].

## Results

Research participants were recruited from all 17 RTOs including nominated representatives of 13. We received 55 valid survey responses, including from seven regional training delegates; 10 CE/CMs; two training posts; and 36 Registrars (Table 2). Interview data were collected from 90 participants representing 13 RTOs, 11 Indigenous Health Training Posts and representatives of 18 other stakeholder organisations (Table 2). Fifty-one participated in face-to- face interviews and 38 in telephone interviews including, in the latter group, representatives of 18 key stakeholder organisations. Fifty- nine participants including CEs, CMs and Medical Educators (some of whom had been previously interviewed) engaged in the large group workshop. As interviews and survey responses were de-identified and not linked prior to analysis, it was not possible to determine how many survey respondents participated also in the interviews. As is clear from Table 2 however, the surveys predominantly sampled Registrars whilst CE/ CM, Medical Educator and Indigenous Health Training Post respondent views were more often accessed through interviews.

Our findings are presented below under the overarching themes of understandings about cultural education and cultural mentoring; the employment and support of CEs and CMs; and delivery and evaluation of cultural education and mentoring.

### Understandings of cultural education and mentoring

**Fig. 1:**
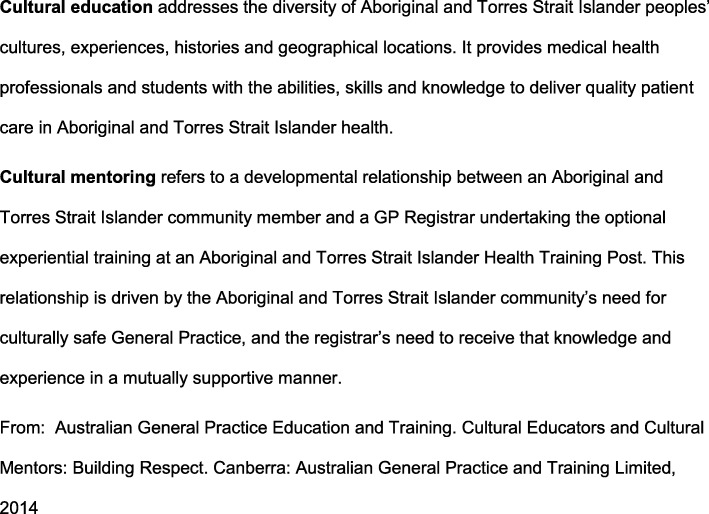
Definitions of Cultural Education and Cultural Mentorship

Participants generally supported definitions of cultural education and mentoring developed in consultation with CEs and CMs (Fig. 1). However the complex nature of learning and teaching about Aboriginal and Torres Strait Islander culture was highlighted, along with need for flexibility in teaching roles.

*… the description is basically close to my own but, I guess it is too cold, too clinical …there should be a sacred sense of mystery about a person’s culture, but how in the world would you pass that on?* (CE/ CM, survey)

*What cultural awareness does and cultural safety does, is give the broader perspective of a completely different outlook that allows us to see that we’re two people in a room, in a consulting room, and your experience may well be hugely different to mine.* (Stakeholder, interview).

A key consideration was respect for the value of Aboriginal and Torres Strait Islander people’s knowledge and intellectual property including that of CEs/CMs and patients.

*Hopefully [Registrars] will grasp the concept that although they wear a ‘white coat’ [this] does not necessarily mean they possess more ‘knowledge’ than that of the Aboriginal patient.* (CE/CM, survey).

*It is important to understand that the intellectual property belongs to the community or the individual and should not be exploited or exported as a right without consent or acknowledgement. (CE/CM, survey).*

Face to face interaction with Aboriginal and Torres Strait Islander people was seen as a critical element in cultural education, which was recommended to include both cultural and historical impacts on health and health care and to be specific to a local context.

*Allow them more time to spend in community visiting people, visiting significant sites and having hands on approach. Having a real cultural immersion experience instead of a watered down classroom experience.* (CE/CM, survey)

*We have this word “Aboriginal” that’s actually a coloniser’s word...that puts one thing onto this hugely disparate group of cultures, and then attached to that, we have cultural education and cultural mentoring as if we can teach this one thing, and we can’t.* (Stakeholder, interview)

Reflection on one’s own culture was considered a key component of cultural learning.

*…it helps create awareness of how the culture of our patient and our own cultures (in all the many aspects) affect our interaction…* (Registrar, survey).

Participants considered cultural education and mentoring delivered different learning experiences. Cultural education was seen to prepare Registrars for work with Aboriginal and Torres Strait Islander individuals and communities, ensuring baseline knowledge. Mentoring involved support of the learner as they worked in a community and was characterised as an ongoing dialogue with both mentor and mentee gaining from the process. From the learner perspective, the mentoring relationship was observed to offer a safe and personalised learning environment.

*[Cultural mentoring] also provides a contact to whom one can direct cultural questions without fear of being culturally insensitive…* (Registrar, survey).

Though the roles of both CEs and CMs were noted to vary according to location, this was particularly characteristic of the CM, described by one CE interviewee as the ‘extended arm (into the community) of the CE’. Interviewees commented that the CM needed to be the ‘right’ person, as cultural mentoring requiring particular personal qualities which can’t always be learned and concern the authority given by the community for the CM to take this role.

Critically, interviewees highlighted the right of the local community and the training post to define CE/CM roles according to local needs and context and recommended avoiding a constrictive focus on terminology.

*I don't want to be a formal Cultural Mentor or Educator because...I don't feel like it is my right to do that when I'm not from here. Might be different if I'm back home but not here…Maybe it’s about the title.* (IHTP Aboriginal staff member, interview)

*Sometimes it’s just around clarifying the role though, I mean demystifying it a bit because … it was never meant to be like … the person who was the owner of all the cultural knowledge stuff. It was just somebody who was Aboriginal and was local, that knew a bit about their local culture that could actually help people out a bit, or even just point them in the right direction.* (CE/CM, interview)

### Employment and support of cultural educators and cultural mentors

All participants valued the CE and CM roles and the vast majority also noted that these roles needed to be undertaken by Aboriginal and Torres Strait Islander people.

However some uncertainty was expressed about who was appropriate to fill the roles and how best to engage and support them. CE and CM roles were reported as filled by a variety of people employed by different organisations, with many providing cultural education or mentoring alongside other substantive roles or, particularly in the case of CMs, in unpaid and sometimes unrecognised positions. The pressure of general practice training on the relatively small Aboriginal and Torres Strait Islander workforce was clearly recognised.

*There’s mentoring going on in* [the Indigenous Health Training Post]*, no question. We know that, but we don’t employ them. It just happens. And they’re not getting paid for it. They’re just a health worker and they have to fit that in with all the other thousand things that’s required of a health worker.* (RTO delegate, interview)

The challenges in defining and setting limits around the role, including for those who combined cultural mentoring with other work roles, increased the complexity of determining appropriate remuneration and employment parameters.

*It [cultural mentoring] happens for nothing at the moment… someone in the community will see that someone is struggling, and … some bond will form with the health worker or an Aboriginal member of the community …That additional role of CM is too much…[training organisations] have to acknowledge that it’s a special set of skills, and that people should be paid for it.* (Stakeholder, interview)

*There’s been great difficulty in sort of trying to set up the notion of the more formalised CM program…theoretically you want the person to be available “all the time”. You can’t pay someone to be available all the time…so what is it that you need them to be able to do?* (Stakeholder, interview)

Interviewees also described challenges related to appointing CMs and the importance of succession planning.

*How to appoint a Cultural Mentor?…well, I think that it’s a bit tricky… it does have to be somebody who’s just generally recognised as being someone who’s able to take on that title… one should be thinking about successors and potential additional people to the pool … if someone appears to be maybe a good person for the role, they should be … maybe mentored by the cultural mentor.* (RTO delegate, interview).

The costs for those undertaking these often unremunerated roles were repeatedly emphasised with cultural mentoring seen to be a challenging role. When roles were not clearly agreed by all parties including CMs, Registrar and RTO expectations could be unrealistic. Participants also described the personal cost for CEs and CMs, which put them at risk of burn out and potentially less effective relationships with Registrars.

*… that relationship can be different and can be pretty dynamic depending on where you are, who you’re working with and who’s giving you the education, or who’s mentoring.* (CE/CM, interview)

*… it must be very hard for an Aboriginal person to do this on an ongoing basis. I have witnessed staff have their personal story questioned and vilified. The ones I see give it their all die just a little every time they run a session, till they say they can do it no more.* (CE/ CM, survey).

Support for CEs and CMs varied greatly with key support strategies including adequate and appropriate remuneration, recognition in terms and conditions of employment, organisational support, and strong partnerships with Medical Educators.

*…remuneration is more than just money. For instance, if you work in a community as a doctor you’re supplied with a house to live in, your electricity is paid, your phone is paid, your Internet is paid, your TV is paid for… Community people…see these people come and go, and have plenty of advantages. It’s not unreasonable, I think, sometimes for them to expect that you might take your car hunting, when it’s provided for you…things like paying the telephone bill for someone who is able to be a CM is a way of saying that you value that they are available on the telephone.* (RTO delegate, interview)

The support of local Aboriginal and Torres Strait Islander communities was critical for CEs and CMs.

*…as an Aboriginal person, I go back to family members. So I speak to my Elders a lot. I suppose I’m pretty lucky, I’m pretty strong in my culture …I’ve been taught a lot.* (CE/CM, interview)

Some recommended recognition of cultural learning and responsibilities in CE/ CM employment terms and conditions.

Few CE/ CM professional development or career advancement opportunities were described. Although not recommended as employment requirements, professional development such as individualised professional development plans, peer mentoring and national networking were suggested to sustain CE/ CM engagement. Other training suggestions included teaching and mentoring skills training, and an understanding of cross-cultural stress and its manifestations in a non-Aboriginal person.

*I would like to see all CEs having access to … a recognised course in Training and Assessment. Many have the cultural knowledge but lack the presentation and assessment skills which could enhance their role. (CE/ CM, survey)*

*How to recognise cultural stress in a non-Aboriginal person, …[health professionals] tend to get quite directive, because the world is not organised how they are used to it, and then they want to organise it; they want to do that, they start trying to organise all the people, and the Aboriginal mob just turn off.* (Stakeholder, interview)

Cultural education skills training also needed to take account of local context and recognise the different learning needs and styles of individuals.

*I don’t think [formal CE training] will work for everyone because we all talk from the heart…if you have to sit them in a classroom it’d be the worst thing possible and I wouldn’t want to do it.* (CE/CM, interview)

A CE/ CM career pathway was recommended to make these roles more attractive, in the same way that medical career pathways were clearly defined.

*People should have the opportunity to travel [on a career pathway] as a cultural mentor, to become a cultural educator, to become a senior cultural educator, and then to become a supervisor.* (CE/CM, interview)

### Delivering and evaluating cultural education and mentoring

Cultural education was reported to be delivered across all training regions though cultural mentoring appeared less widely available, occurring mostly in training posts.

*I feel that, in general, cultural education was done well but did not know much about cultural mentoring.* (Registrar, survey).

For some participants however cultural education was also not prioritised sufficiently. Few RTOs extended cultural education to staff and to GP Supervisors. CEs were more often engaged in delivery of programs than in development and evaluation. Evaluative processes could be perfunctory.

*…As a result of some black box of cultural education, cultural mentoring… we’re producing doctors that patients go, “Oh, these guys I can trust.” And then it would lovely to open up the lid of a black box and say, “Okay, what are the components of this that we know produce those outcomes?” At the moment, all we’re asking is, “Are you happy?” …but actually, some of the most effective education won’t make them happy, it will make them uncomfortable and question what they’re doing, because we’ve got it wrong for 200 years.* (Training post GP Supervisor, interview)

Interviewees described the ideal of cultural competence as a lifelong process supported at organisational level and shaped by continuous cultural education, experience and evaluation and assessment. Assessment, particularly of values and attitudes, was not widely reported and this was observed to make tailoring of cultural education to the individual Registrar difficult. The importance of involving Aboriginal and Torres Strait Islander people in evaluation and assessment was clearly highlighted.

*Patients need to be asked and communities, “Is this doctor any good? Can you trust them? Can you trust them more after this intervention that we’ve done?” And that’s really hard and quite a long term thing to be able to measure, so it’s not done, but that’s what we need to demonstrate.* (Training post GP supervisor, interview)

Integration of cultural and clinical teaching was recommended and noted to be supported by respectful partnerships between CEs/ CMs and Medical Educators as well as supportive structures within RTOs.

*…integrates the cultural education with clinical education* i.e. *cultural aspects which can impact on the consultation…* (RTO delegate, survey).

## Discussion

Aboriginal and Torres Strait Islander people working as CEs and CMs have a central role in face-to-face education of general practice Registrars about local culture and history and related impacts on health. This role was highly valued by all our participants. Whilst cultural education was described as providing base-line learning in preparation for practice with Aboriginal and Torres Strait Islander patients, cultural mentoring was seen by our participants as longitudinal, relationship-based learning and was particularly valued by Registrars working in Aboriginal and Torres Strait Islander communities, though less frequently provided compared to cultural education.

Yet there were observed to be challenges in providing this model of training. A key challenge described by our participants is finding people with the skills and authority to undertaken the demanding work inherent to CE and CM roles. Remuneration is also a challenge, particularly for CMs whose work-time is difficult to quantify and who are often employed in other substantive roles and sometimes not paid at all. Appropriate remuneration and employment terms and conditions, provision of relevant professional development, strong partnerships with Medical Educators, as well as organisational support were identified as important for sustaining CE and CM roles.

The focus in Australia on health care provider education regarding Indigenous cultures is shared with many other countries [7, 20] although the evidence for efficacy of cultural education in terms of improving health outcomes or enhancing access to health care is limited [4, 21]. In alignment with the literature, our participants described the ideal of respectful engagement in life-long learning that required self-reflection on the part of the learner and monitoring and support at organisation levels [22–24].

Addressing this ideal, recent international reviews of cultural diversity training report a range of strategies, including tuition by those whose culture is the subject of the teaching and immersion in communities [8, 20, 25]. However there is no mention of ongoing engagement of educators in roles similar to CE/CM roles beyond the Australian general practice training context. The network of CEs and CMs who provided guidance for our research has drafted definitions of the CE and CM roles (Fig. 1).

Our research, prioritising the voices of those engaged in these roles, focusses on challenges encountered in delivering this model of training and makes a number of recommendations for the Australian general practice training context which are likely to be relevant to those looking for sustainable approaches to delivery of cultural competency training for healthcare providers in other countries and settings.

CEs and CMs described a lack of training and confidence and poor health as well as personal costs in re-living the trauma recounted in teaching which have been described previously [14, 16]. Our research also documents organisational challenges in identifying people and occasional reticence in taking the title of CE or CM, as well as overlap between the roles. Providing those identified by the community as appropriately skilled and with the authority to teach about these important issues, with a role or job description, rather than assigning them a narrow title, may facilitate a more flexible approach. This may assist Indigenous educators to be able to move between education and mentoring, and assist those already working in other substantive roles to take on additional educational responsibilities within existing roles. There are parallels with roles such as the “health coach” recommended in enhanced primary health care, in that this role can be filled by a variety of professionals who may transition over time to different roles in the health system [26].

Although a focus on roles over titles could assist in flexibility, lack of a title may hamper promotional opportunities in non-Indigenous organisations and compromise career pathways for these experienced educators. This was a concern for our interviewees as was the lack of professional development for CEs and CMs. Suggestions for professional development included skills and knowledge in medical educational approaches, however context was noted to be important with training such as in use of technologically based educational approaches, unlikely to be culturally appropriate for all CE/CMs, whilst remote and on-line approaches may be useful for others. There is clearly a need to respect and value Indigenous teaching approaches such as story- telling which have also been recommended in both research and clinical environments [27].

This need for respect infuses much of our data. It includes respectful relationships with learners and with Medical Educators and organisational respect for the CEs and CMs. Calls for organisational support for cultural education from our participants echo similar advocacy in the international literature for systems approaches to improving cultural competence in healthcare [20, 23]. The culture of training organisations has a powerful influence on the learner, transmitting values and attitudes and shaping behaviours [28–30]. The absence of a rigorous theory of the role of culture in medicine [31] can contribute to perceptions that cultural competence is ‘unscientific’ [32, 33] with cultural education an “afterthought” in medical training [15]. Our interviewees spoke about equally valuing cultural education and medical teaching as a “two-way learning approach” [16].

Organisational respect was seen to include wider support for Aboriginal and Torres Strait Islander peoples such as Reconciliation Action Plans which aim to make non-Indigenous organisations a safe place for Aboriginal and Torres Strait Islander people to work [34]. Respectful partnerships between training organisations and local communities not only ensure training is appropriate to that community but together with strong leadership, create a shared responsibility for sustained change [35]. The reflexivity essential for learners to become culturally competent [15] could equally well be prescribed for organisations engaging in this work.

In a cycle perhaps reflective of the cultural context of this research, the CEs and CMs for whom the organisation needs to be culturally safe can assist both in developing this capacity for organisational critical reflection, as well as establishing and maintaining connections to communities that will sustain change over time.

### Strengths and weaknesses of the research

A key strength of this research has been the leadership and guidance by CEs and CMs from inception, as well as within the research team and overseeing the research. Whilst this strong engagement with those whose experience was under investigation may be considered by some to be a conflict of interest, it also assisted us in gaining the trust of participants and provided a sound understanding of the research context as well as ensuring the research was culturally appropriate. The wide range of participants both in terms of roles and geography was a unique feature of our research. Though survey respondents were unlikely to constitute a representative sample of the various stakeholder groups, we found substantial concordance between qualitative survey responses and interviews.

## Conclusions

Our research, undertaken in an Australian general practice training context, reflects on the unique roles of CEs and CMs in this context and provides a valuable opportunity to learn from those engaged in these roles. Their reflections highlight the importance of CE and CM roles as well as challenges in sustaining them. The recommendations of our participants concerning professional and organisational support of those engaged in this work, align with the international literature and provide valuable insights for those seeking to develop respectful and effective cultural education programs.

## Abbreviations

CE: Cultural educator
CM: Cultural mentor
GP: General practitioner
RTO: Regional training organisation
