# Correction to: Supporting Aboriginal and Torres Strait islander cultural educators and cultural mentors in Australian general practice education

**DOI:** 10.1186/s12909-018-1359-z

**Published:** 2018-11-22

**Authors:** Jennifer Reath, Penelope Abbott, Linda Kurti, Ruth Morgan, Mary Martin, Ada Parry, Elaine Gordon, Julian Thomas, Marlene Drysdale

**Affiliations:** 1 Department of General Practice, Western Sydney University, Campbelltown, Australia; 2 Department of General Practice, Western Sydney University, Sydney, Australia; 3 URBIS, Sydney, Australia; 4 Western Sydney University, Sydney, Australia; 5 Queensland Aboriginal and Islander Health Council, South Brisbane, Australia; 6 Menzies School of Health Research, Darwin, Australia; 7 Murray City Country Coast GP Training, Melbourne, Australia

## Correction

Following publication of the original article [1], the author reported that Fig. 1 was missing. The correct version of Fig. 1 is given below. This error was introduced during the production process, and the original article has been corrected.

**Fig. 1:**
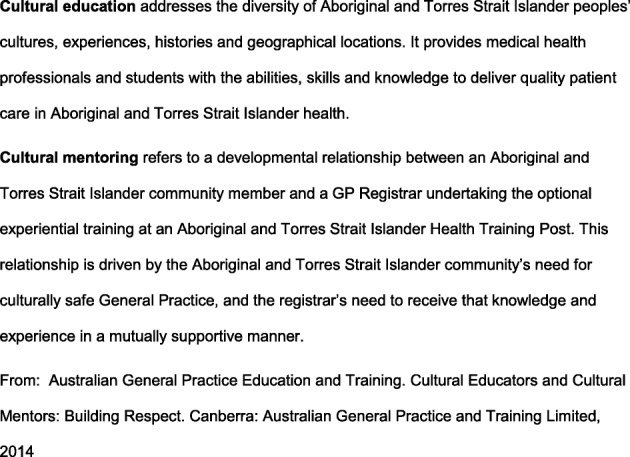
Definitions of Cultural Education and Cultural Mentorship

## References

[1] Reath J (2018). Supporting aboriginal and Torres Strait islander cultural educators and cultural mentors in Australian general practice education. BMC Med Educ.

